# Touch imprint cytology is useful for the intraoperative pathological diagnosis of PitNETs’ surgical margins

**DOI:** 10.1007/s10014-023-00470-9

**Published:** 2023-10-06

**Authors:** Noriaki Tanabe, Naoko Inoshita, Atsushi Ishida, Masataka Kato, Haruko Yoshimoto, Hideki Shiramizu, Hidetaka Suga, Toru Tateno, Kenichi Ohashi, Shozo Yamada

**Affiliations:** 1 Department of Pathology, Moriyama Memorial Hospital, Tokyo, 134-0081 Japan; 2 Department of Human Pathology, Tokyo Medical and Dental University, Tokyo, Japan; 3 Department of Neurosurgery, Japan Self Defense Forces Hospital, Tokyo, Japan; 4 Department of Endocrinology and Diabetes, Nagoya University Graduate School of Medicine, Nagoya, Japan; 5 Division of Endocrinology and Metabolism, Department of Medicine, University of Alberta, Edmonton, AB Canada; 6 Hypothalamic & Pituitary Center, Moriyama Neurological Center Hospital, Tokyo, Japan

**Keywords:** Pituitary neuroendocrine tumor, PitNET, Intraoperative pathological diagnosis, Surgical margin, Touch imprint cytology

## Abstract

Touch imprint cytology (TIC) and frozen section (FS) procedures are essential for intraoperative pathological diagnosis (IPD). They are invaluable tools for therapeutic decision-making, helping surgeons avoid under or overtreatment of patients. Pituitary neuroendocrine tumors (PitNETs) are generally small, slow-growing tumors with low-grade malignancy located at the base of the skull where it is impossible to maintain a wide tumor margin. Therefore, transsphenoidal surgery (TSS) should be performed with necessary caution, and with sufficient and minimal resection. Thus, this study aimed to evaluate the diagnostic accuracy of TIC for the diagnosis of PitNET and determine its ability to accurately evaluate the surgical margin compared to the FS procedure. A total of 104 fresh specimens from 28 patients who underwent TSS for PitNETs were examined using TIC and FS. TIC specimens were categorized according to the cell imprinting pattern. All specimens with a large number of neuroendocrine cells diffusely attached to the glass surfaces had PitNET components. Contrarily, no rich or diffuse cell attachments were observed in any non-tumoral endocrine cells. In conclusion, recognizing a pattern of endocrine cell adherence to glass is highly effective in IPD to certify the existence of a PitNET component.

## Introduction

Pituitary neuroendocrine tumors (PitNETs), formerly referred to as pituitary adenomas, are clinically classified into two groups: (1) functional PitNETs with hormonal disorders and (2) non-functional tumors with locally compressive and invasive symptoms, such as visual disturbances and headaches. PitNETs are low-grade malignant neuroendocrine tumors located at the base of the skull, where it is nearly impossible to maintain a wide tumor margin. As a result, transsphenoidal surgery (TSS) must be performed with necessary caution ensuring sufficient but minimal resection.

In general, intraoperative pathological diagnosis (IPD) has two main purposes: (1) to determine tumor existence and its pathological type and (2) to evaluate the tumor boundary for complete resection. Touch imprint cytology (TIC) and frozen section (FS) procedures are crucial for IPD [1]. For pituitary tumors, the FS procedures require pathologists who are highly skilled in specimen processing and diagnosis due to the small size of the specimen (average, 1–2 mm), and occasionally, cell morphology cannot be evaluated due to artifacts caused by the freezing process [1, 2]. Therefore, for pituitary tumors, cytology is sometimes more effective than FS for IPD, especially in determining the histological subtypes and hormone-producing activity [2–4]. The latest atlas from the Armed Forces Institute of Pathology (AFIP) mentions that cytology is not useful for surgical margin determination because it does not provide information on tissue architecture [5]. However, our team has been performing marginal evaluation using IPD for PitNETs and has used both FS procedures to examine architecture and TIC techniques to examine cell characteristics and attachment patterns in all routine IPDs for the past several years. Using a combination of these techniques, we achieved higher accuracy rates compared to those achieved using a single technique.

Adrenocorticotropic hormone (ACTH) producing PitNETs, which cause Cushing disease, may be difficult to detect using imaging. Kurosaki et al. reported that intraoperative cytology is highly accurate in detecting the presence of PitNETs [6]. However, in previous reports, while cytology was used to evaluate tumor presence, it has rarely been used to evaluate surgical margins. In other organs, TIC performed alone replaces the FS process to evaluate the surgical margins. For example, the evaluation of TIC for sentinel lymph nodes in breast cancer has been shown to be effective [7]. During partial nephrectomy for renal tumors, marginal evaluation with TIC is highly accurate in determining whether partial resection is acceptable [8].

Although we already use a combination of TIC and FS for IPD in our clinical practice routinely, in this study, we aimed to determine the accuracy of TIC compared to FS for PitNET diagnosis and the evaluation of surgical margins.

## Materials and methods

A total of 104 fresh specimens from 28 patients who underwent TSS for PitNETs between April and June 2022 at Moriyama Memorial Hospital were examined for intraoperative pathological consultation. Our clinical team always uses IPD to decide regarding clinically effective resection. All cases had one specimen obtained from the clinically central part of the tumor (clT) to identify the histological variation of the tumors and subsequently, other specimens from clinically surgical margins (clSM), which were defined as pseudo-membranous tissue composed of PitNET and compressed anterior lobe tissue, or from sites where the surgeons could not certify whether tumor components existed, sometimes with irregular fibrosis.

**Fig. 1:**
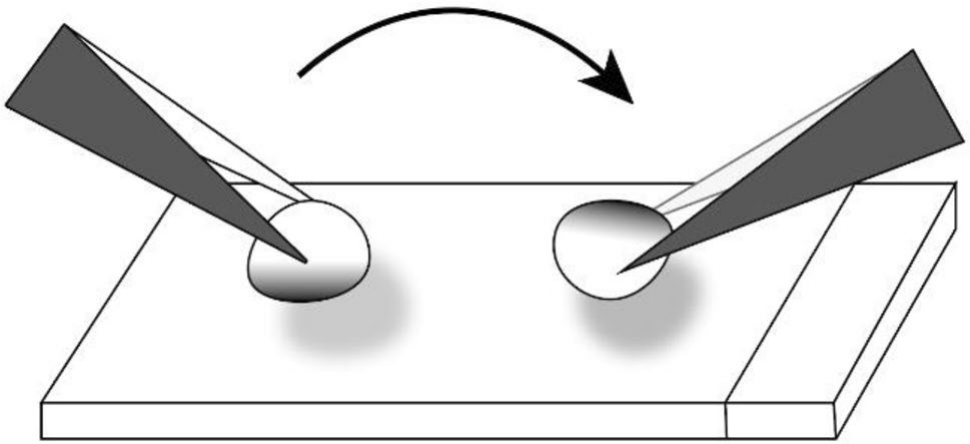
In our touch imprint cytology method, we rolled the specimens and touched two or more surfaces with the microscope glass slides

Coated glass slides (MAS-01; Matsunami Glass Industry, Osaka, Japan) were used for both the TIC and the FS procedure. At first, small and fresh tissue samples retrieved during TSS were touched onto dry microscopic glass slides for imprint preparation, and the slides were immediately fixed with 95% alcohol. During the imprinting procedure, at least two surfaces were attached and rolled to examine circumferentially (Fig. 1). After cytology processing, the specimens were mounted in a compound (Tissue Tek O.C.T Compound; Sakura Finetek, Tokyo, Japan), rapidly frozen in isopentane chilled by dry ice, and sectioned at 5 µm. The specimens from both the TIC and FS procedures were stained using the rapid hematoxylin and eosin procedure, dehydrated, permeabilized, and sealed with a glass cover. After the FS procedure, the specimens, excluding the pure tumors for deep freeze storage, were re-fixed with formalin and processed into paraffin-embedded sections for re-diagnosis.

All slides were independently examined and discussed by a pathological trainee (NT) and a specialized pathologist (NI) with experience in diagnosing over 3,000 PitNET cases.

**Fig. 2:**
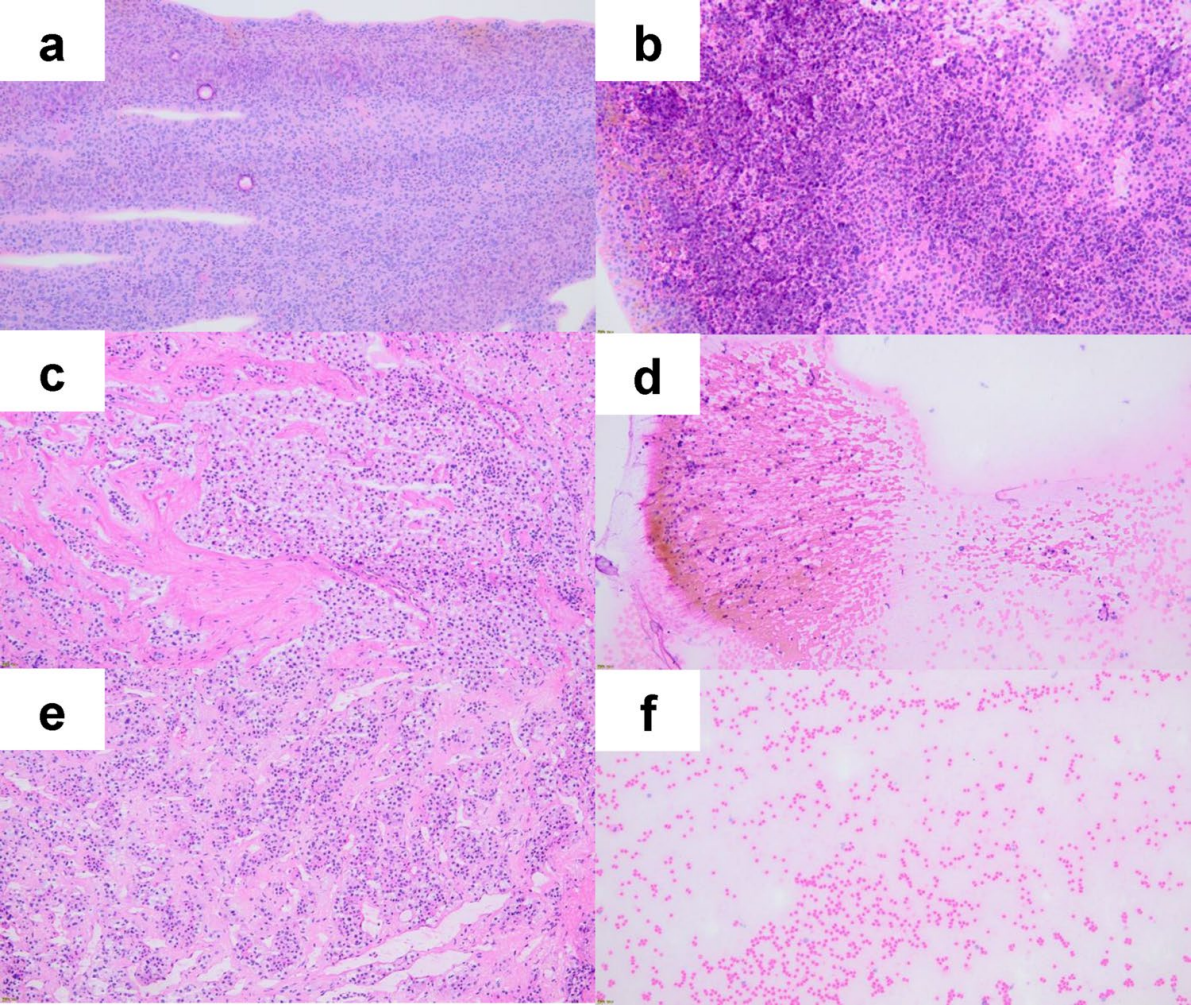
**a**–**f** Microscopic images of hematoxylin and eosin staining of touch imprint cytology preparation and frozen section. All specimens were obtained with × 10 magnification. Both **a** and **b** were from the same specimen from hT. **c** and **d** were from hB. **e** and **f** were from hN. **a**, **c**, and **e** were from frozen section, and **b**, **d**, and **f** were touch imprint cytology and classified as follows; **b** as cyA1, **d** as cyB1, and **f** as cyC

**Fig. 3:**
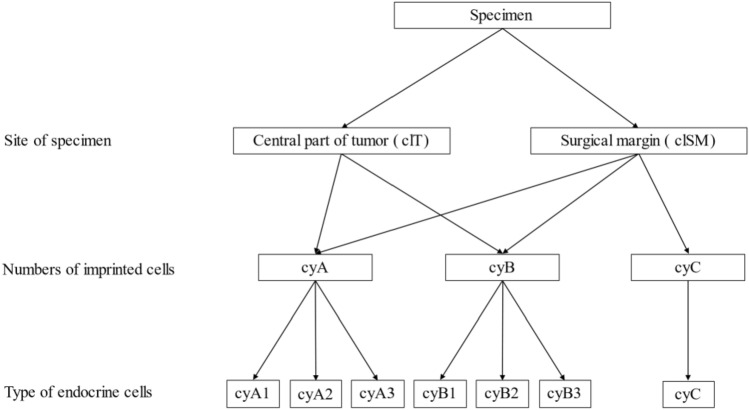
A breakdown of cytological classification of specimens

The cytological findings were classified into the following three categories according to the cell imprint pattern (Fig. 2): pattern cyA, rich and diffusely spread neuroendocrine cells (Fig. 2b); pattern cyB, a small number of scattered endocrine cells (Fig. 2d); and pattern C, no apparent endocrine cells (Fig. 2f). Patterns cyA and cyB were further classified into three subcategories based on the morphology of the endocrine cells: 1; PitNETs, 2; adenohypophyseal cells, and 3; difficult to determine (Fig. 3).

Histologically, with FS on the site of IPD, each specimen was divided into one of the three groups: “histologically tumor (hT)” (tumor component only), “histologically boundary (hB)” (both PitNET and a normal component), and “histologically normal (hN)” (no tumor component). Subsequently, they were evaluated histologically using the formalin re-fixed specimens after FS. The diagnostic accuracy of TIC was validated based on histological findings using both frozen and formalin re-fixed specimens for the final diagnosis.

Based on these definitions, the positivity, sensitivity, specificity, positive predictive value, negative predictive value, and accuracy of TIC were evaluated. The Kappa coefficient was used for diagnostic concordance between TIC and FS histology. Statistical analyses were performed using EZR ver. 1.54 [9]. The Kappa coefficient was assessed per Koch et al.’s definition [10]. Although cyA1 and cyB1 were cytological PitNET-positive specimens, the possibility that a small number of PitNET cells in the surgical field were attached to the surface of the specimen could not be ruled out in cyB1. Therefore, we raised two statistical questions that defined only cyA1 as cytologically positive and both cyA1 and cyB1 as cytologically positive.

## Results

**Fig. 4:**
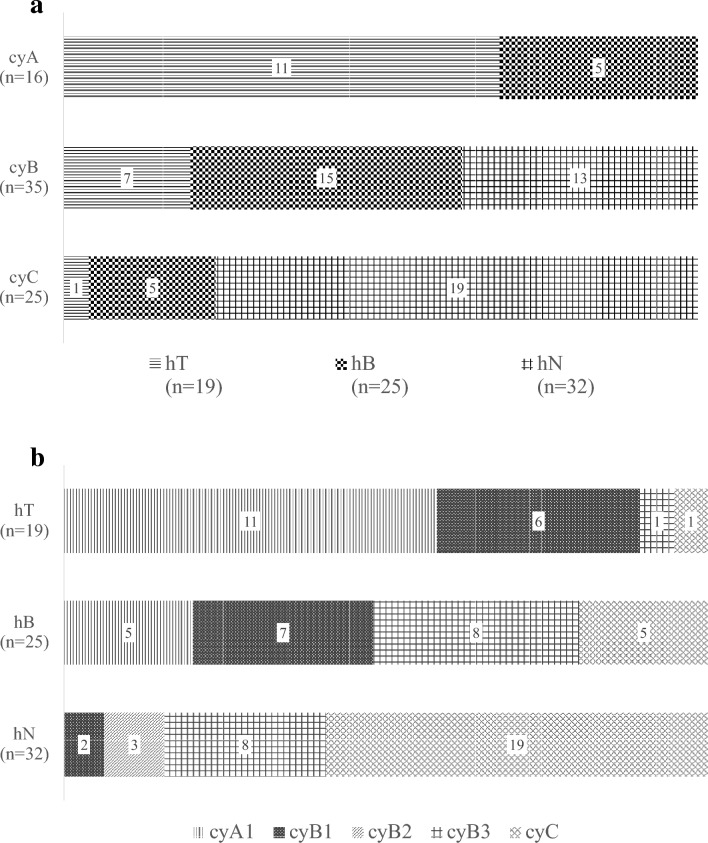
**a** Histological diagnosis distributions of the each touch imprint cytological pattern from surgical margins. **b** Composition of touch imprint cytology pattern varied from sample site

The results of the cytological evaluations are presented in Table 1. Tumor types of these cases were classified as follows: thirteen gonadotroph PitNETs, four somatotroph PitNETs, four lactotroph PitNETs, three corticotroph PitNETs, two thyrotroph PitNETs, an immature PIT1- lineage tumor, and a mammosomatotroph tumor. All 28 cytological specimens from clT comprised 26 specimens of cyA1 and 2 specimens of cyB1. Regarding clSM, the final histological diagnoses by FS and formalin re-fixed sections were 19 hT, 25 hB, and 32 hN, among a total of 76 specimens. Among the hN specimens, 13 cases (40.6%) had neuroendocrine cells and 2 cases (6.2%) were determined to be cyB1; positive cytologically. However, none of the TIC specimens of the hN type were classified as pattern cyA (Fig. 4a).

**Table 1:** Distribution of the cytological pattern according to the histological evaluation

	Cytological pattern^*1^						
	cyA1	cyA2	cyA3	cyB1	cyB2	cyB3	cyC
Histological diagnosis^*2^							
cl T^*3^							
hT^*4^ (*n* = 28)	26	0	0	2	0	0	0
cl SM^*3^							
hT^*4^ (*n* = 19)	11	0	0	6	0	1	1
hB^*4^ (*n* = 25)	5	0	0	7	0	8	5
hN^*4^ (*n* = 32)	0	0	0	2	3	8	19

*PitNET* pituitary neuroendocrine tumor, *cy* cytological, *cl* clinical, *h* histological, *T* tumor,

*SM* surgical margin, *B* boundary, *N* normal

*1 Cytological pattern: cyA1, rich monomorphous tumoral cells; cyA2, rich polymorphous endocrine cells; cyA3, rich undeterminable cells; cyB1, sparse monomorphous tumoral cells; cyB2, sparse polymorphous endocrine cells; cyB3, sparse undeterminable cells; cyC, no apparent endocrine cells

*2 Performed using frozen sections and FFPE sections of the same specimens, except the pure tumor sample for deep freeze storage

*3 clT, specimens from the center of the tumor; cl SM, specimens for surgical margin evaluation

*4 hT, histologically containing tumor only; hB, histologically containing both tumor and anterior lobe tissue; hN, histologically containing no tumor component

cyA1 + cyB1, defined as cytologically positive, had a sensitivity of 61.4%, specificity of 93.8%, and positive predictive value of 93.1%. Assuming that only cyA1 was positive, the sensitivity was 36.4%; however, the specificity and positive predictive values were 100%. When comparing the accuracy between the two groups, cyA1 + cyB1 was significantly more accurate (*P* < 0.001). The Kappa coefficient for A1 as positive was 0.32 and that for cyA1 + cyB1 as positive was 0.52 (Table 2).

**Table 2:** Test accuracy with two different definitions of positive cytological diagnosis

	cyA1 as positive	cyA1 + cyB1 as positive
Sensitivity	36.4 (22.4–52.2)	61.4 (45.5–75.6)
Specificity	100 (84.2–100)	93.8 (79.2–99.2)
PPV	100 (71.3–100)	93.1 (77.2–99.2)
NPV	53.3 (40.0–66.3)	63.8 (48.5–77.3)
Accuracy	63.2 (51.3–73.9)	75.0 (63.7–84.2)
κ coefficient	0.32 (0.13–0.52)	0.52 (0.33–0.71)

*NPV* negative predictive value, *PPV* positive predictive value

Data are presented as *n* (%) otherwise indicated

In all specimens, pattern cyA were determined to be from samples that contained the tumor, and notably, no cyA2 or cyA3 were present (Fig. 4b). In addition, 17 of the 19 hT specimens in clSM appeared as cyA1 and cyB1, suggesting that we possibly only touched the normal surface of the boundary to the glass and that touching the glass slides with many sides of the specimen is imperative.

**Fig. 5:**
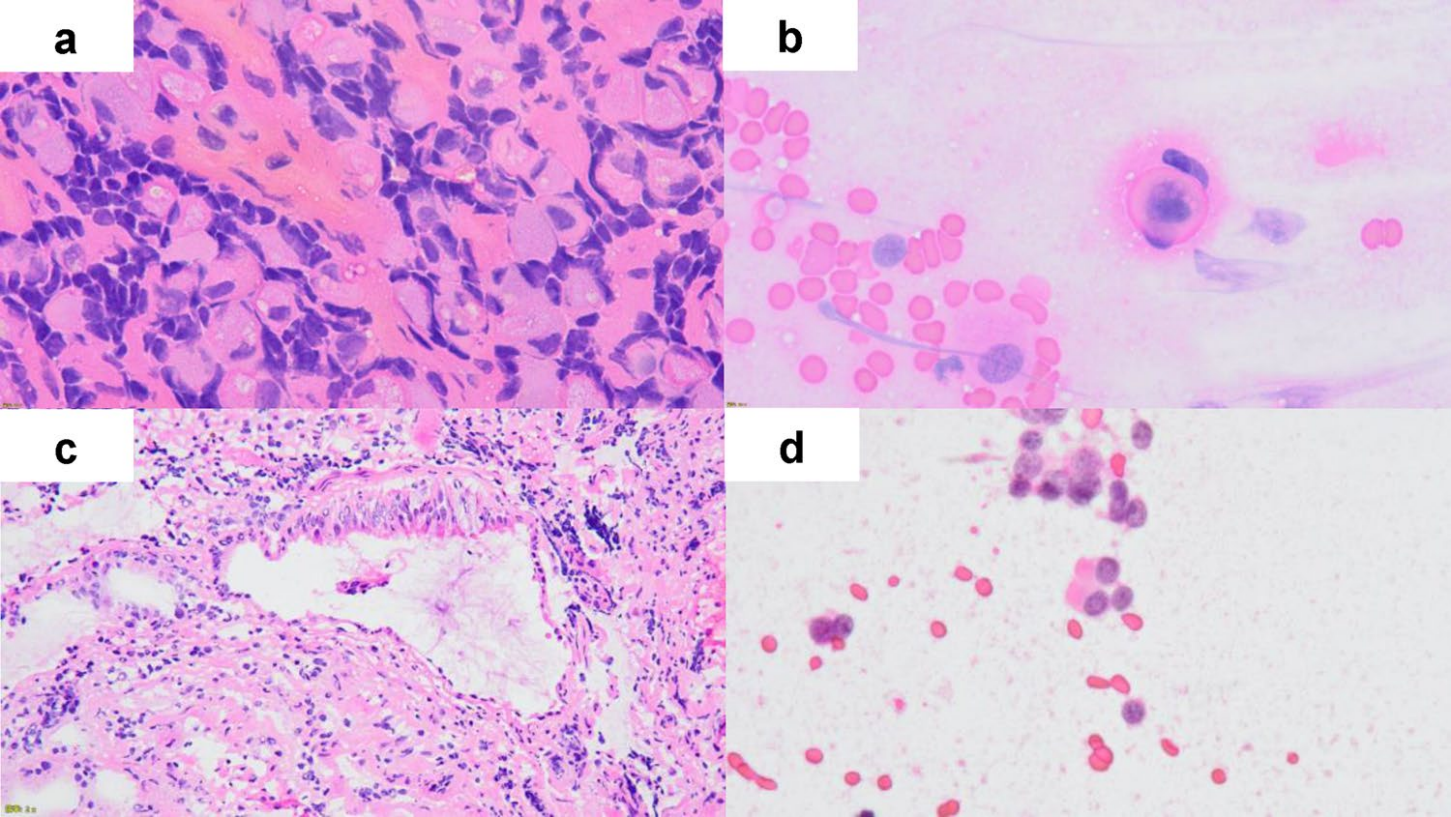
**a** Frozen section of the Crooke hyaline change × 40, **b** its touch imprint cytology of (**a**) × 60. **c** FS of the cyst covered by columnar epithelial cells near PitNET × 20, and **d** its TIC of columnar cells with cilia × 60

Interesting findings were observed in some cases. In one case of corticotroph PitNET, TIC of the hN specimens had relatively many non-tumoral cohesive endocrine cells. We found scattered bizarre large cells with Crooke’s hyaline change, a well-known morphological change of non-tumoral corticotroph cells under the systemic condition of hypercortisolism, and then, we could classify it as cyB2 (Fig. 5a, b). In other cases, the ciliated columnar epithelial cells of the Rathke’s cleft cyst and small retention cysts surrounding PitNETs were difficult to distinguish from neuroendocrine cells. Columnar cells were similar to endocrine cells, especially in nuclear size, and were difficult to distinguish without cilia (Fig. 5c, d).

## Discussion

TIC is easier to prepare for intraoperative consultation than the FS procedure, which requires highly trained and skilled pathological technicians to prepare sufficient glass slides for accurate diagnosis [11]. Our study demonstrated that the diagnostic accuracy of TIC was equivalent to that of the FS procedure in IPD for PitNETs’ surgical margins.

Generally, TIC is considered acceptable for the rapid diagnosis of brain tumors when compared to histological diagnosis [1, 12]. Although a previous report examined the diagnostic concordance rate from the tumor, the present study showed 100% concordance at the macroscopical tumor level, with 92.9% of specimens being determined to be cyA1. A previous report that compared the positive diagnosis rate between touch and squash preparation, reported that TIC was useful for small round cell tumors, such as PitNETs and lymphomas [12]. This study also indicated that TIC was accurate.

**Fig. 6:**
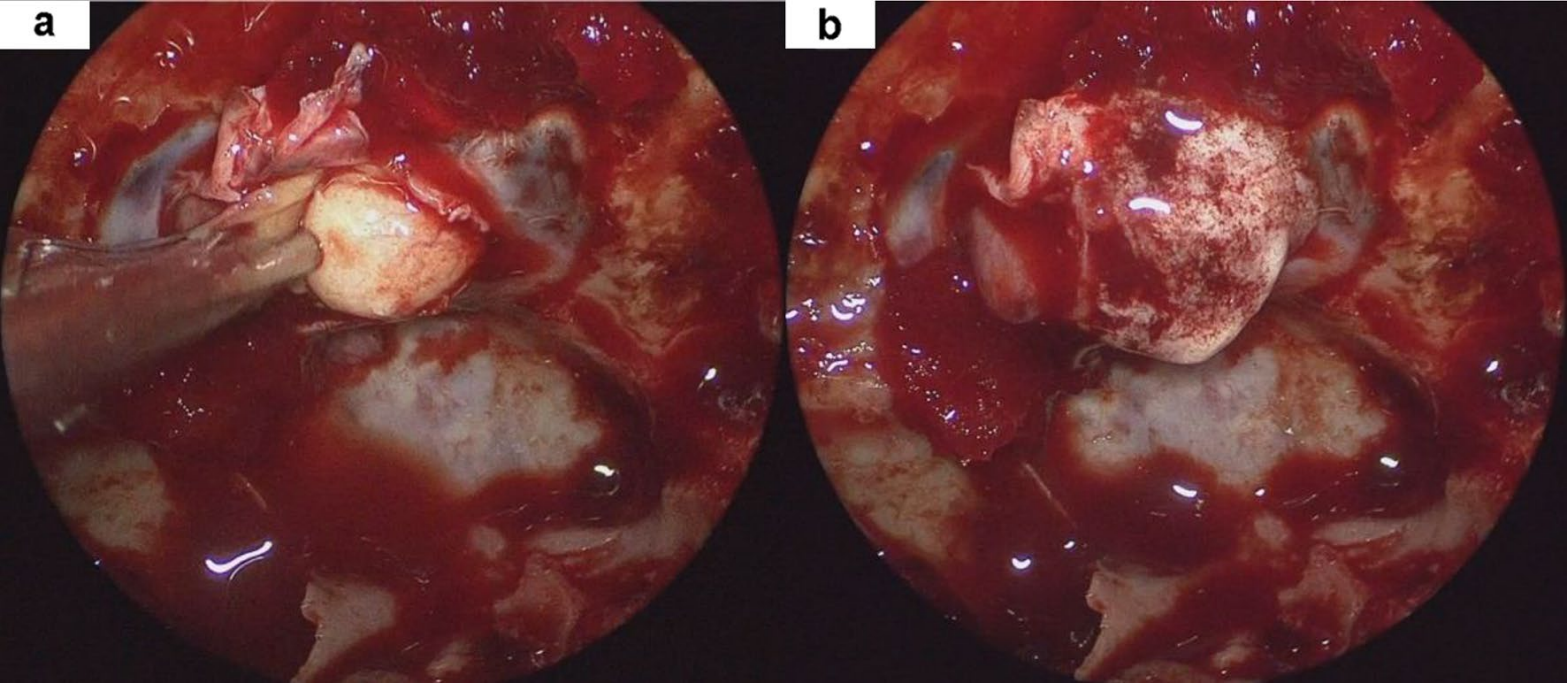
Intraoperative endoscopic findings in representative cases. **a** encapsulated tumor is exposed. **b** tumor fragments floating in the blood during tumor resection

Therefore, it is important to clearly define “cytological positivity.” In this study, diagnosing “cyA1 + cyB1” as positive had a significantly higher accuracy than diagnosing only “cyA1” as positive. In contrast, there was 100% specificity and positive predictive value when only cyA1 was defined as positive. cyA1 was positive for many imprinted endocrine cells without considering whether these cells were tumoral because cyA2 and cyA3 were not present. Although, the reason for the difference in the number of imprinted cells on the TIC glasses is unclear, we estimate that PitNET cells tend to be easily imprinted. The histological distinguishing point of PitNETs from anterior lobe tissue is the breaking of anterior lobe trabecular structure. In fact, during surgery, when the PitNET’s capsule breaks, a whitish tumor component flow out (Fig. 6). If specimens are soaked in saline, PitNET cells may wash out. Therefore, we recommend surgeons place specimens on water-repellent sheets without soaking them in a saline solution and submit them immediately after removal. Then, it can be concluded that the specimens are PitNETs when many small round cells are observed on the glass slides.

Although two cases of cyB1 appeared to be over-diagnosed with a negative judgment by frozen and re-formalin-fixed sections, it was estimated that true PitNET cells on the surface of hN specimens, which could contaminate the operation area, were observed.

Crooke’s hyaline change is a reversible degeneration of corticotroph cells that occurs when exposed to sustained high cortisol levels [13]. In the present study, we observed many imprinted non-tumoral Crooke cells. Thus, the presence of Crooke cells may have loosened the trabecular structure of the anterior lobe. The combination of TIC and FS for IPD for TSS has been routinely performed by our team for several years, and data were partly obtained from many serial cases.

When the frozen and re-formalin fixed sections provided the absolute correct diagnosis, TIC had an accuracy of 75% in the margin evaluation and a Kappa coefficient of 0.52. FS is difficult to evaluate because of the presence of freezing artifacts. In fact, in this study, 10.5% of specimens that were evaluated using the FS procedure were difficult to diagnose. TIC has shown significant promise and avoids artifacts produced by freezing; thus, TIC could be a reliable intraoperative consultation aid.

In conclusion, the diagnostic accuracy of TIC was equivalent to that of the FS procedure for intraoperative pathological consultation of PitNETs’ surgical margins. 

## Data Availability

The datasets generated and analyzed during the current study are available from the corresponding author on reasonable request.
